# The Application of Point of Care Ultrasound to Screen for Pulmonary Hypertension: A Narrative Review

**DOI:** 10.24908/pocus.v9i1.17494

**Published:** 2024-04-22

**Authors:** Danny Yu Jia Ke, Melissa Tso, Amer M Johri

**Affiliations:** 1 Queen's University School of Medicine Kingston, ON Canada; 2 Kingston Health Sciences Centre Kingston, ON Canada

**Keywords:** Pulmonary hypertension, Point of care ultrasound, Pulmonary arterial hypertension

## Abstract

Background: Pulmonary Hypertension (PH) is a condition with several cardiopulmonary etiologies that has the potential of progressing to right heart failure without proper intervention. After a history, physical exam, and investigations, cases of suspected PH typically undergo imaging via a transthoracic echocardiogram (TTE). This is a resource-intensive procedure that is less accessible in remote communities. However, point of care ultrasound (POCUS), a portable ultrasound administered at the bedside, has potential to aid in the diagnostic process of PH. Methods: The MEDLINE, Embase, and CENTRAL databases were searched to screen the intersection of POCUS and PH. Studies involved adult patients, and only English articles were accepted. Reviews, case reports, unfinished research, and conference abstracts were excluded. Our aim was to identify primary studies that correlated POCUS scan results and additional clinical findings related to PH. Results: Nine studies were included after our search. In these studies, POCUS was effective in identifying dilatation of inferior vena cava (IVC); internal jugular vein (IJV); and hepatic, portal, and intrarenal veins in patients with PH. The presence of pericardial effusion, pleural effusion, or b-lines on POCUS are also associated with PH. Conclusions: This review suggests important potential for the use of POCUS in the initial screening of PH. IVC and basic cardiopulmonary POCUS exams are key for PH screening in patients with dyspnea. Right-heart dilatation can be visualized, and peripheral veins may be scanned based on clinical suspicion. POCUS offers screening as an extension of a physical exam, with direct visualization of cardiac morphology. However, more studies are required to develop a statistically validated POCUS exam for PH diagnosis. More studies should also be conducted at the primary-care level to evaluate the value of screening using POCUS for PH in less-differentiated patients.

## Introduction

Pulmonary hypertension (PH) is a severe condition arising from increased pulmonary vascular resistance with complications including right heart (RH) failure and hypoxia 1. Pulmonary Arterial Hypertension (PAH), also known as primary PH, has no other cardiopulmonary etiology, and its 3-year mortality can be as high as 21% 2. Therefore, early screening and detection is an important component of PH management.

There are five types of PH, labeled groups 1-5 by the World Health Organization (WHO) 3, 4 as follows: PAH, PH due to left heart disease, PH due to hypoxia and lung disease, chronic thromboembolic pulmonary hypertension (CTEPH), and PH with unclear or multifactorial mechanisms, respectively. Group 2 PH comprises 68% of PH cases, as reported by Brown et al. 5. Group 5 is the second most common, making up 15%. Group 3 forms 9% and Groups 1 and 4 account for 3% and 2%, respectively 4, 5. Within PAH, there are several subtypes which include idiopathic, heritable conditions associated with PAH such as connective tissue diseases, human immunodeficiency virus infection, portal hypertension, congenital heart disease, schistosomiasis, PAH associated with drugs and toxins, PAH with features of venous/capillary involvement, and persistent PH of the newborn. Though relatively rare, PH is increasing in incidence around the world and becoming a greater healthcare burden 4, 6.

The diagnosis of PH begins with a clinical suspicion, often in patients with dyspnea, reduced exercise tolerance, fatigue, edema, chest pain, and syncope or near-syncope 7. Following clinical suspicion, a thorough cardiac and abdominal physical exam is indicated. After an electrocardiogram and bloodwork, the next step in the workup is typically a trans-thoracic echocardiogram (TTE) 8, 9. Access to echocardiography can pose a barrier to rapid early diagnosis, depending on resource availability such as requiring specialized personnel and equipment 10, 11, 12. Furthermore, echocardiography may be less accessible in some remote communities since these facilities are typically situated in urban centers 13.

However, with the advent of point of care ultrasound (POCUS), ultrasound scans are now employed at the bedside. Images may be obtained quickly and cheaply, allowing for efficient examination and extension of the physical examination that may point to etiologies such as PH contributing to patient symptoms.

**Figure 1:**
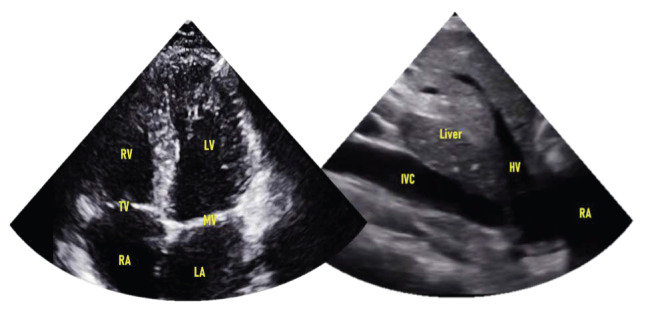
Images of normal cardiac and subcostal views obtainable using a point of care ultrasound (POCUS) examination. The following heart and abdominal structures are labeled: Right and left ventricles (RV, LV), tricuspid and mitral valves (TV, MV), right and left atria (RA, LA), inferior vena cava (IVC), liver,and hepatic vein (HV).

Currently, PH is often missed, especially in younger patients using existing bedside approaches to history and physical examination 5. Even in secondary PH, there is still the risk of RH failure in addition to the primary disease. The increased efficiency of a cardiopulmonary POCUS exam could motivate a broader differential diagnosis that includes PH. Relevant cardiovascular images that apply to PH are shown in Figure 1. This article reviews the potential for POCUS to aid in the diagnostic process of PH and discuss its implications.

## Methods

We conducted a literature review using the MEDLINE, Embase, and CENTRAL databases, capturing the intersection of POCUS and PH using these terms, their synonyms, and relevant subject headings. The full search criteria are shown in the Supplementary Material. Only English studies involving adult patients were included. Conference abstracts, reviews, unfinished research, and case studies were excluded. After title and abstracts were screened, included articles underwent full-text screening. Data from relevant articles were extracted. One reviewer [D.K.] performed the entire screening and extraction.

## Results

**Figure 2:**
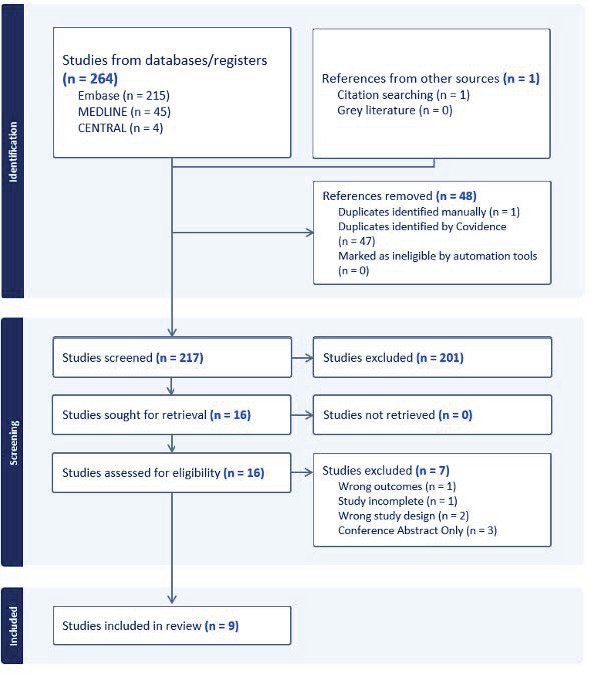
The PRISMA diagram of the resulting literature search. Nine studies were included in this review.

We found nine studies that correlate POCUS findings with PH-related clinical features. The PRISMA diagram is displayed in Figure 2. The results are summarized in Table 1.

Samant et al. 14 qualitatively estimated the right atrial pressure (RAP) using POCUS by measuring IVC size and collapsibility. PH patients (60% Group I) were then stratified into normal, intermediate, and high eRAP. BNP values (in pg/ml) were 70 (95% Confidence interval [CI] 39–120), 166 (CI 80–341), 236 (CI 111–503), respectively. Differences of normal vs intermediate and normal vs high eRAP groups were both significant at p<0.01.

Avriel et al. 15 studied the effect of POCUS in a PAH clinic. 36 patients were randomized 1:1 to either receive an additional POCUS assessment or not. The number of changes to patients’ management was recorded. Cardiac, pulmonary, and IVC POCUS exams were performed. There were 48 management changes in the POCUS group and 18 in the control group (p<0.001).

Torres-Arrese et al. 16 used POCUS to scan acute heart failure patients’ lungs and their hepatic, portal, intrarenal, and femoral veins, both on admission and discharge. The congestion values of the hepatic, portal, and intrarenal veins were used to calculate a Venous Excess Ultrasound Score (VExUS), estimating the congestion in the venous system 17. In paired analysis for patients’ results on admission and discharge, the researchers found that the score significantly correlated with the EVEREST score, a validated clinical scoring for congestion 18, with a correlation of 0.532 (p=0.004). The VExUS also correlated with a decrease in NT-proBNP, with a correlation of −0.411 (p = 0.024).

Dayioglu et al. 19 employed POCUS to measure the following cardiopulmonary parameters, comparing them to conventional ultrasound: fractionated area change; IVC collapsibility/distensibility; LV/RV end-diastolic and IVC diameter; LV/RV diastolic and systolic centricity index; right atrium area; pulmonary artery diameter. None of these parameters were significantly different from conventional handheld ultrasound. Overall, POCUS was 68% accurate for detecting PH with 88% negative predictive value.

Kurnik et al. 20 analyzed POCUS performed on admission to ICU in patients >70 years-old with COVID-19 pneumonia. Pulmonary artery systolic pressure (PASP) was significantly higher in non-survivors compared to survivors (40.4 vs 32.5 mmHg, p = 0.024). Furthermore, a greater percentage of non-survivors had diffuse lung b-lines compared to survivors (59% vs 33%, p = 0.005).

Elzeneini et al. 21 compared POCUS RAP, estimated from the IVC scan, to that determined by RHC. They found a correlation of 0.80, P<.001, and that POCUS was 76-92% accurate overall.

Chopra et al. 22 found that in PH patients, pericardial effusion was 89% predictive of systemic venous hypertension (RAP > 10mg at rest), while pleural effusion had only a 67% positive predictive value for pulmonary venous hypertension (pulmonary artery wedge pressure > 15mmHg). Both effusions were found using POCUS.

Parikh et al. 23 used POCUS to measure the internal jugular vein (IJV) in patients undergoing RHC for PH. The following were measured: IJV diameter at rest, during respiratory variation, and during manual compression. The collapsibility indices were calculated during respiration and during manual compression. The measured RAP correlated significantly with the IJV diameter (r=0.26, p=0.029). Furthermore, the compression collapsibility index correlated significantly with mRAP (r=-0.43, p=0.0002), pulmonary artery occlusion pressure also obtained from an RHC (r=-0.35, p<0.0027), and BNP levels (r=-0.31, p=0.015).

Simon et al. 24 studied patients undergoing RHC, but not necessarily for suspected PH. The right IJV cross-sectional area (CSA) with and without the Valsalva maneuver was measured using POCUS. Patients were grouped as normal and elevated mRAPs. The Valsalva maneuver increased IJV CSA by a median of 35% (IQR 19%-79%) in normal mRAP patients, and 5% in high mRAP patients (IQR 3%-14%). They reported that a >17% increase in right IJV CSA with Valsalva can predict elevated RAP with a sensitivity and specificity of 90% and 74%, respectively.

**Table 1:** Results of the review. Five primary studies evaluating point of care ultrasound (POCUS) were found relevant to pulmonary hypertension (PH). The number of patients (n), patient population, and clinical findings are also summarized.

**Study ID**	**n**	**Population**	**POCUS Technique**	**Measured using POCUS**	**Correlated significantly with**	**Ref**
Samant 2022	90	PH, 60% Group I	IVC - Subcostal view	RAP - IVC diameter and collapsibility	BNP levels	14
Avriel 2023	36	PAH (Group I)	PLAX, PSAX, A(5,4,2)C, SC, IVC. Lung congestion, b-lines, pneumothorax, atelectasis, pleural effusion, consolidations	Use of cardiopulmonary exams, IVC diameter	Management changes, attributed to POCUS use	15
Torres-Arrese 2022	30	PH in Acute Heart Failure (Group II PH)	Peripheral venous scan	Hepatic, portal, and intrarenal vein congestion, combined into the VExUS score	NT-proBNP and EVEREST grading score	16
Dayioglu 2024	46	Cardiopulmonary problems, at high risk for PH	PLAX, PSAX, A4C, A4CRV, SC, IVC, RA using B mode	Cardiopulmonary measurements	The same on conventional ultrasound; no significant differences between them	19
Kurnik 2023	117	Age >70 in ICU for COVID-19 pneumonia	Lung exam with 8 areas + cardiac exam.	PASP and presence of pleural b-lines	Death	20
Elzeneini 2022	50	PH patients undergoing RHC	IVC - Subcostal view in sagittal plane	RAP - IVC diameter and collapsibility	RAP using RHC	21
Chopra 2021	32	PH patients undergoing RHC	PLAX, PSAX, A4C, SC4C, abdomen/thorax	Presence of pleural effusion and pericardial effusion	Systemic and pulmonary venous hypertension from RHC	22
Parikh 2019	71	PH patients undergoing RHC	Linear probe, with and without compression. Color doppler implemented	IJV diameter	RAP using RHC	23
Simon 2010	67	Patients undergoing RHC	Head turned left, supine. Probe at right sternocleidomastoid	Right IJV cross-sectional area with and without valsalva	RAP using RHC	24
POCUS = point of care ultrasound; PH = pulmonary hypertension; PAH = pulmonary arterial hypertension; RHC = right heart catheterization; BNP = b-type natriuretic peptide; NT = N-terminal; PLAX = parasternal long axis; PSAX = parasternal short axis; A_C = apical _-chamber; SC4C = subcostal 4-chamber; PASP = pulmonary artery systolic pressure; RAP = right atrial pressure; VExUS = Venous Excess Ultrasound; EVEREST = Efficacy of Vasopressin Antagonism in Heart Failure: Outcome Study with Tolvaptan						

## Discussion

POCUS is an inexpensive, fast, and accessible tool that is increasingly prevalent in outpatient settings. POCUS examinations have the potential to be used as a screening tool for PH. The POCUS exams used to screen for PH are: parasternal long axis (PLAX), parasternal short axis (PSAX), apical 4-chamber (A4C), and subcostal 4-chamber (S4C), subcostal for the IVC and estimating RAP, and B-mode pulmonary views of each lobe. These techniques are derived from ultrasound guidelines and are standard across included studies 25, 26, 27. The full cardiopulmonary POCUS exam and veins encompassing the IVC and peripheral veins are all reasonable in the screening of PH, as outlined below.

The IVC diameter and collapsibility arising from the subcostal view has been the most used and validated estimate of the RAP among the studies, and it should be the preferred screening method for PH on POCUS.

The cardiac exam uses standard visualizations PLAX, PSAX, A4C, and S4C. The goal is to identify pericardial effusion, RH dilatation, tricuspid regurgitation, and rule out other cardiac etiologies such as left heart or valvular diseases. RH dilatation was not thoroughly investigated in the studies included, as it arises later in the disease process of PH 28, 29. Meanwhile, tricuspid regurgitation is a complication of RH dilatation and can also be viewed on POCUS 30, 31.

The pulmonary exam employs B-mode investigating each lobe, and visualizes the presence of b-lines and pleural effusion. These are associated with PH based on our results. In addition, the pulmonary exam scans for signs of pulmonary decompensation while ruling out other pulmonary causes of the presentation.

The IJV may be useful to obtain RAP. However, the IVC has been more thoroughly studied in the studies we reviewed. Other peripheral veins such as the hepatic, portal, and intrarenal veins are also effective proxies for the RAP. However, they are distal to the IVC and should only be examined under exceptional circumstances or with complaints such as peripheral swelling.

**Figure 3:**
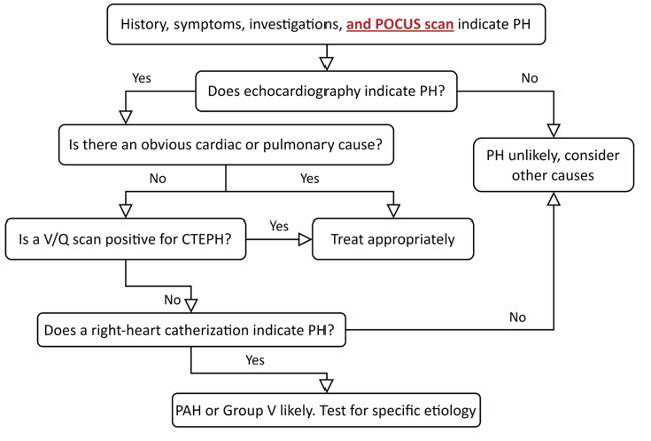
The proposed addition of point of care ultrasound (POCUS) to the initial screening for pulmonary hypertension (PH). Echocardiography, a ventilation/perfusion (V/Q) scan, and a gold-standard right-heart catheterization (RHC)are still required to progress in the diagnosis as needed should the PH probability be high. CTEPH = Chronic thromboembolic pulmonary hypertension.

**Figure 4:**
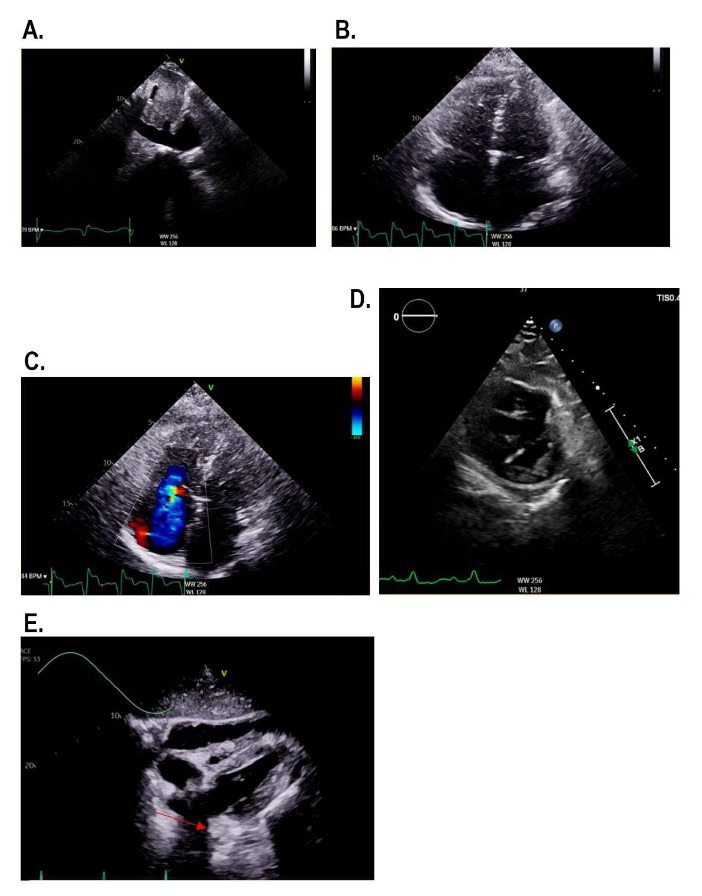
Abnormal point of care ultrasound (POCUS) scans of patients with PH A. Inferior Vena Cava (IVC) Dilatation. B. Right Ventricle (RV) Dilatation. C. Severe Tricuspid Regurgitation. D. Dilated RV in the parasternal short axis. The D-shaped septum is indicative of RV pressure overload. E. Pericardial effusion.

A summary of the POCUS exam for PH screening is outlined in Table 2. The screening role of POCUS in the diagnostic algorithm of PH is outlined in Figure 3. Select abnormal POCUS findings are pictured in Figure 4.

**Table 2:** Summary of POCUS exam for PH screening, illustrating techniques used and the exam’s objectives

**POCUS Exam**	**POCUS technique(s) used**	**Exam objective(s)**
Inferior Vena Cava	Subcostal	Diameter and collapsibility to estimate RAP
Cardiac Exam	PLAX, PSAX, A4C, SC4C	Pericardial effusion, RH dilatation, rule out other cardiac etiologies
Pulmonary Exam	B-mode exam of each lobe	B-lines, pneumothorax, atelectasis, pleural effusion, consolidations
Internal jugular vein*	Longitudinal and cross-sectional visualization	Internal jugular vein diameter
Peripheral Venous Scan*	Longitudinal and cross-sectional visualization	Hepatic, portal, and intrarenal vein diameter
*only need to be performed for unclear findings or clinical suspicion of peripheral decompensation. PLAX = parasternal long axis; PSAX = parasternal short axis; A4C = apical 4-chamber; SC4C = subcostal 4-chamber; RAP = right atrial pressure; RH = right heart		

There are several limitations of this study. First, while RHC is the gold-standard for the diagnosis of PH, it is utilized less due to its invasiveness. Therefore, other clinical findings indicative of PH such as lab values and clinical scores were also used. This makes it is difficult to isolate the effect of the PH itself in a patient complicated by other cardiopulmonary disease unless the focus was only on patients with PAH. Another limitation was the heterogeneity and overall low number of studies in this field. The studies are unable to be amalgamated due to differences in patient population, group of PH, experimental design, regions investigated by POCUS, and outcomes examined.

Therefore, the goal for the future is to produce a fast and clinically validated screening exam utilizing current standards. This screening exam would be intended for patients in the primary care setting who present with dyspnea and other PH-related symptoms, and would ultimately serve as an extension of the physical exam. To that end, more studies are required in this field, to determine exact measurements, their cutoffs for severity, and their relative weights of consideration. These studies should be replicated to allow for the creation of more statistically robust POCUS exams for PH.

In addition, more studies should be conducted in a primary care setting. None of the studies reviewed focus on undifferentiated patients presenting with dyspnea and how POCUS can help screen them for PH. These studies would be more similar to their proposed use in the context of PH. While the studies that were reviewed illustrated the ability for POCUS to estimate severity in cohorts of PH patients, undifferentiated patients at an earlier stage of disease may present with less discernible findings. Other presentations and differential diagnoses in a primary care setting may also add complexity to the protocols outlined in these studies.

Nonetheless, POCUS should still be used today to help screen for PH where indicated. Even though the absence of positive findings on POCUS cannot rule out PH, the presence of findings can further the probability of PH and the acute risk to the patient. This information can be used to triage limited space and resources in the echocardiography lab, and to communicate the urgency of such a procedure. This information would still be of great importance in rural and remote communities, where transportation to an echocardiography lab may be less accessible.

## Conclusion

POCUS can supplement the physical exam during the screening for PH. Compared to echocardiography, POCUS is also more accessible in outpatient and rural/remote areas. We find that POCUS for PH should primarily involve the IVC. A cardiac and pulmonary exam are also indicated to look for RH dilatation, pleural and pericardial effusions, b-lines, and other cardiopulmonary comorbidities. Other peripheral veins such as the IJV, hepatic, portal, and intrarenal veins may be assessed if clinical suspicion is present for peripheral vascular decompensation. Basic cardiopulmonary POCUS techniques are standard across studies we reviewed, but a standardized and validated POCUS exam for PH still needs to be developed. To that end, more studies are required in both PH and primary care populations. The use of POCUS to screen for PH is still in its infancy, and this article provides a foundation on which future research can build. Nevertheless, POCUS can still serve an important role in triaging, maximizing limited echocardiographic resources, and more accurately estimating a patient’s urgency in rural and remote areas. 

## Disclosures

None.

## Supplementary Material

Appendix A Full online search strategy.
