# Supplementary material for: The Application of Point of Care Ultrasound to Screen for Pulmonary Hypertension: A Narrative Review
Source: POCUS J. 2024 Apr 22;9(1):109–16. doi: 10.24908/pocus.v9i1.17494 (PMC11044931; doi:10.24908/pocus.v9i1.17494)
Supplement: Appendix A [file pocusj-09-17494-s001.pdf]

## Supplementary

### *Search Strategies, performed Jan 29, 2024:*

MEDLINE

| | |
|---|---|
| 1 | pocus.mp. |
| 2 | handheld ultrasound.mp. |
| 3 | point of care ultrasound.mp. |
| 4 | point of care ultrasonography.mp. |
| 5 | bedside ultrasound.mp. |
| 6 | 1 or 2 or 3 or 4 or 5 |
| 7 | pulmonary hypertension.mp. |
| 8 | pah.mp. |
| 9 | pulmonary arterial hypertension.mp. |
| 10 | hypertension, pulmonary/ or familial primary pulmonary hypertension/ or pulmonary arterial hypertension |
| 11 | 7 or 8 or 9 or 10 |
| 12 | 6 and 11 |

Embase

| | |
|---|---|
| 1 | pocus.mp. |
| 2 | handheld ultrasound.mp. |
| 3 | point of care ultrasound.mp. |
| 4 | point of care ultrasonography.mp. |
| 5 | bedside ultrasound.mp. |
| 6 | exp "point of care ultrasound"/ |
| 7 | 1 or 2 or 3 or 4 or 5 or 6 |
| 8 | pulmonary hypertension.mp. |
| 9 | pah.mp. |
| 10 | pulmonary arterial hypertension.mp. |

11 exp “pulmonary hypertension”/

12 8 or 9 or 10 or 11

13 7 and 12

## CENTRAL

1 pocus.mp.

2 handheld ultrasound.mp.

3 point of care ultrasound.mp.

4 point of care ultrasonography.mp.

5 bedside ultrasound.mp.

6 1 or 2 or 3 or 4 or 5

7 pulmonary hypertension.mp.

8 pah.mp.

9 pulmonary arterial hypertension.mp.

10 hypertension, pulmonary/ or familial primary pulmonary hypertension/ or pulmonary arterial hypertension

11 7 or 8 or 9 or 10

12 6 and 11
